# Actos Now for the prevention of diabetes (ACT NOW) study

**DOI:** 10.1186/1472-6823-9-17

**Published:** 2009-07-29

**Authors:** Ralph A DeFronzo, MaryAnn Banerji, George A Bray, Thomas A Buchanan, Stephen Clement, Robert R Henry, Abbas E Kitabchi, Sunder Mudaliar, Nicolas Musi, Robert Ratner, Peter D Reaven, Dawn Schwenke, Frankie B Stentz, Devjit Tripathy

**Affiliations:** 1 Texas Diabetes Institute and University of Texas Health Science Center, San Antonio, TX, USA; 2 Suny Health Science Center at Brooklyn, Brooklyn, NY, USA; 3 Pennington Biomedical Research Center/LSU, Baton Rouge, LA, USA; 4 University of Southern California Keck School of Medicine, Los Angeles, CA, USA; 5 Division of Endocrinology & Metabolism, Georgetown University, Washington, DC, USA; 6 VA San Diego Healthcare System and University of California at San Diego, USA; 7 University of Tennessee, Division of Endocrinology, Diabetes and Metabolism, Memphis, TN, USA; 8 Medstar Research Institute, Hyattsville, MD, USA; 9 Phoenix VA Health Care System, Phoenix, AZ and W.P. Carey School of Business, Arizona State University, Tempe, AZ, USA

## Abstract

**Background:**

Impaired glucose tolerance (IGT) is a prediabetic state. If IGT can be prevented from progressing to overt diabetes, hyperglycemia-related complications can be avoided. The purpose of the present study was to examine whether pioglitazone (ACTOS^®^) can prevent progression of IGT to type 2 diabetes mellitus (T2DM) in a prospective randomized, double blind, placebo controlled trial.

**Methods/Design:**

602 IGT subjects were identified with OGTT (2-hour plasma glucose = 140–199 mg/dl). In addition, IGT subjects were required to have FPG = 95–125 mg/dl and at least one other high risk characteristic. Prior to randomization all subjects had measurement of ankle-arm blood pressure, systolic/diastolic blood pressure, HbA_1C_, lipid profile and a subset had frequently sampled intravenous glucose tolerance test (FSIVGTT), DEXA, and ultrasound determination of carotid intima-media thickness (IMT). Following this, subjects were randomized to receive pioglitazone (45 mg/day) or placebo, and returned every 2–3 months for FPG determination and annually for OGTT. Repeat carotid IMT measurement was performed at 18 months and study end. Recruitment took place over 24 months, and subjects were followed for an additional 24 months. At study end (48 months) or at time of diagnosis of diabetes the OGTT, FSIVGTT, DEXA, carotid IMT, and all other measurements were repeated.

Primary endpoint is conversion of IGT to T2DM based upon FPG ≥ 126 or 2-hour PG ≥ 200 mg/dl. Secondary endpoints include whether pioglitazone can: (i) improve glycemic control (ii) enhance insulin sensitivity, (iii) augment beta cell function, (iv) improve risk factors for cardiovascular disease, (v) cause regression/slow progression of carotid IMT, (vi) revert newly diagnosed diabetes to normal glucose tolerance.

**Conclusion:**

ACT NOW is designed to determine if pioglitazone can prevent/delay progression to diabetes in high risk IGT subjects, and to define the mechanisms (improved insulin sensitivity and/or enhanced beta cell function) via which pioglitazone exerts its beneficial effect on glucose metabolism to prevent/delay onset of T2DM.

**Trial Registration:**

*clinical trials*.gov identifier: NCT00220961

## Background

Type 2 diabetes mellitus (T2DM) affects 21 million Americans [1] and approximately 194 million individuals world wide [2] and its prevalence is rapidly increasing [3]. Microvascular [4] and macrovascular [5] complications are common in T2DM and both are strongly related to the severity and duration of hyperglycemia [6-8]. The estimated cost for the treatment of T2DM and related complications in year 2007 was 174 billion dollars [9].

The natural history of T2DM has been well defined [10-14], starting with a genetic predisposition and progression from normal glucose tolerance with insulin resistance to impaired glucose tolerance with superimposition of beta cell failure on insulin resistance, and eventually to overt T2DM characterized by severe beta cell failure and insulin resistance.

Because of the central role that hyperglycemia plays in the development of both micro- and macrovascular complications [4-8], it follows that interventions designed to prevent or delay the onset of hyperglycemia would be effective in preventing these long term complications. Recent studies have demonstrated that life style modification [15,16] and therapy with metformin [16] and thiazolidinediones [14,17,18] can prevent or delay the onset of T2DM in high risk subjects. Individuals with impaired glucose tolerance (IGT) represent a high risk group, with a conversion rate that varies from 3–13% per year, depending upon the ethnic group [19].

Individuals with IGT are characterized by defects in both insulin secretion and insulin sensitivity [20-22]. Therefore, interventions designed to enhance beta cell function and ameliorate insulin resistance would be expected to be effective in preventing the progression of IGT to T2DM. Pioglitazone and other thiazolidinediones have been shown both to enhance muscle/hepatic/adipocyte insulin sensitivity [23-26] and to improve beta cell function [16,18,27,28]. Because thiazolidinediones reverse the characteristic defects present in IGT and T2DM, they represent a logical choice to prevent/delay the onset of diabetes in high risk individuals.

## Methods/Design

### Primary Objective

The primary purpose is to examine whether treatment of individuals with IGT with pioglitazone can prevent or delay the development of T2DM.

### Secondary Objectives

Secondary objectives include whether pioglitazone can: (i) improve glycemic control; (ii) enhance insulin sensitivity; (iii) improve beta cell function; (iv) improve risk factors for cardiovascular disease; (v) cause regression/slow progression of carotid intima-media thickness, (vi) decrease microalbuminura; (vii) revert newly diagnosed type 2 diabetic subjects to a state of normal glucose tolerance.

### Overall Study Design/Subject Eligibility

The study is a prospective, randomized, double-blind placebo controlled trial to examine the efficacy of pioglitazone in reducing the incidence of T2DM in adults (≥18 years) with IGT, as defined by a 2-hour plasma glucose concentration of 140–199 mg/dl during a single OGTT [29].

In addition, all IGT subjects must have a fasting plasma glucose of 95–125 mg/dl and at least one of the following: (i) one or more components of the insulin resistance syndrome (low HDL cholesterol [<40 mg/dl in females; <35 mg/dl in males], fasting plasma triglyceride ≥ 150 mg/dl, sitting blood pressure > 135/85 mmHg or on active therapy for hypertension, BMI ≥ 25 kg/m^2 ^[BMI ≥ 22 kg/m^2 ^for Asian Americans], waist circumference > 102 cm in men and > 88 cm in women [>90 cm in Asian American men and > 80 cm in Asian American women]), (ii) family history of type 2 diabetes mellitus (≥1 first degree relatives); (iii) history of gestational diabetes mellitus; (iv) polycystic ovarian syndrome; (v) minority ethnic background (Mexican American, African American, Asian, Pacific Islander, Native American). The inclusion criteria included: (i) men and women ≥ 18 years of age, (ii) all ethnic groups, (iii) IGT with FPG = 95–125 mg/dl plus at least one high risk characteristic; IGT is defined as a 2-hour plasma glucose = 140–199 mg/dl during a single 75 gram OGTT; (iv) BMI ≥ 25 kg/m^2 ^(no upper limit) (BMI > 22 for Asian Americans). Exclusion criteria are presented in the Table 1.

**Table 1:** Inclusion/Exclusion Criteria.

• ***1. Inclusion Criteria***
• Men and women
• All ethnic groups
• ≥ 18 years of age
• IGT with a FPG = 95–125 mg/dl plus at least one additional high risk
• characteristic (see text). IGT is defined as a two hour plasma glucose
• concentration = 140–199 mg/dl during a single 75 gram OGTT.
• BMI ≥ 25 kg/m^2 ^(no upper limit) (BMI > 22 kg/m^2 ^for Asian Americans)
***1. Exclusion Criteria***
• Subjects with diabetes mellitus: FPG ≥ 126 mg/dl or 2-hour plasma glucose ≥ 200 mg/dl during OGTT.
• Subjects previously treated with a thiazolidinedione (ever) or metformin (within one year prior to randomization)
• Subjects previously treated with a sulfonylurea, a meglitinide, an alpha glucosidase inhibitor for more than one week within the last year, or within the 3 months prior to randomization
• Subjects previously treated with insulin (other than during pregnancy) for more than one week within the last year or within the 3 months prior randomization.
• Medical conditions likely to limit life span and/or increase risk of intervention
- Cardiovascular disease
- Hospitalization for treatment of heart disease or stroke in past 6 months
- New York Heart Association Functional Class > 2
- Left bundle branch block or third degree AV block
- Aortic stenosis
- Systolic blood pressure > 180 mmHg or diastolic blood pressure > 105 mmHg; subjects can be re-screened after treatment of their hypertension
- Renal disease (creatine ≥ 1.6 mg/dl for men or ≥ 1.5 mg/dl for women, or urine protein ≥ 2+)
- Anemia (hematocrit < 33% in men and < 30% in women); if the hematocrit increases above these levels at a later date, they can be included in the study
• Hepatitis, based on history and/or serum ALT greater than 2.5 times the upper limit of normal
- Other gastrointestinal disease (pancreatitis, inflammatory bowel disease)
- Recent or significant abdominal surgery
- Pulmonary disease with dependence on oxygen or daily use of bronchodilators
- Chronic infection (e.g., HIV, active tuberculosis)
• Conditions or behaviors likely to affect conduct of the trial
- Unwilling to accept treatment assignment by randomization
- Participation in another intervention research project that might interfere with completion of the study
- Weight loss of > 10% in past 6 months for any reason except postpartum weight loss
- Currently pregnant or within 3 months postpartum
- Currently nursing or within 6 weeks of having completed nursing
- Pregnancy anticipated during the course of the trial
- Unwilling to undergo pregnancy testing or report possible pregnancy promptly
- Unwilling to take precautions to avoid pregnancy if potentially fertile
• Major psychiatric disorders
• Excessive alcohol intake, either acute or chronic
• Medications and medical conditions likely to confound the assessment for diabetes, including:
Thiazide diuretics at a dose greater than 25 mg/day
Non-cardioselective beta-blockers (individuals receiving treatment with a statin or fenofibrate will not be excluded as long as the dose has been stable for 3 months prior to randomization)
Glucocorticoids, systemic
Prescription weight-loss or weight-gain medications
• Thyroid disease, suboptimally treated as indicated by abnormal serum thyroid-stimulating hormone
• Other endocrine disorders (e.g. Cushing's syndrome, acromegaly)
• Fasting plasma triglyceride > 400 mg/dl, despite treatment
• Individuals with a history of bladder cancer
• Individuals with hematuria at screening. However, subjects with hematuria may be randomized if the cause of the hematuria is found, treated, and thought unlikely to recur.

During the course of recruitment, the investigators agreed to modify the glycemic inclusion criteria to allow enrollment of persons with fasting glucose of 90–125 mg/dl as long as the 2-hour plasma glucose concentration was 170–199 mg/dl, in recognition of the high risk of diabetes in such persons.

Eight centers took part in the study and the protocol was approved by the individual IRB of the 8 participating centers. After ascertaining eligibility and collection of baseline measures as described below, subjects were randomized by center and gender using block randomization to ensure equal distribution. Prior to randomization all subjects met with a dietician and received 30 minutes of instruction consistent with the goals of the Diabetes Prevention Program [16]. These goals emphasized reduced total caloric intake, decreased fat intake, and walking 30 minutes per day for 4–5 days per week. Goals were reinforced on all follow up visits.

Potentially eligible IGT subjects (n = 1850) were identified according to recruitment procedures most suited to each of the 8 participating centers and their written, voluntary informed consent was obtained. Subjects then received a 75 gram oral glucose tolerance test (OGTT) with samples drawn at -30, -15, 0, and every 15 minutes thereafter for 2 hours for determination of plasma glucose, insulin, C-peptide, and free fatty acid concentrations. The OGTT was performed at 0800 h following an overnight fast (after 2000 h). Samples for determination of plasma glucose concentration were sent to the Central Laboratory (Texas Diabetes Institute, San Antonio, TX) and sites were notified about subject eligibility (FPG ≥ 95 mg/dl and 2-h PG = 140–199 mg/dl) within 48 hours. Subjects meeting these criteria returned for a medical history, physical examination, screening blood tests (chemistries and complete blood cell count), urinalysis, and electrocardiogram. During this visit or on a subsequent visit prior to randomization, a fasting blood sample was obtained for the measurement of HbA_1c_, and lipid profile, and plasma and serum were collected for later measurement of novel risk factors. The first voided morning urine specimen was collected for determination of microalbumin to creatinine ratio and a 24-hour urine was collected for determination of 8-epi-prostaglandin F_2_α (also termed 15-F_2t_-IsoP), a measure of oxidative stress [30]. Systolic and diastolic blood pressures were measured with an automated Dinamap Pro 100 instrument (GE Medical Systems, Milwaukee, WI) following 5 minutes in the reclining position and after 5 minutes in the upright position. Ankle-arm blood pressure was measured as described by Papamichael et al [31].

Body composition was assessed by measurement of body weight (to the nearest 0.1 kg) on a digital scale (Health-O-Meter, Bridgeview, IL) and height (to the nearest 0.1 cm). Waist circumference was measured using the Gulick II Tape Measure, model #67010 (Gays Mills, WI) at the midpoint between the highest point at the iliac crest and the lowest part of the costal margin in the midaxillary line. Waist circumference was measured in the morning in the erect position after voiding and following an overnight fast. Percent body fat and lean body mass were determined by DEXA using the 4500A Hologic (Bedford, MA) in 4 of the 8 centers. For quality assurance, a Spine Phantom (Hologic) was scanned every morning and a Step Phantom (Hologic) was scanned every week. Standardization and cross-validation of the DEXA measurement between the 4 participating centers was achieved by performing 5 consecutive (once daily) measurements using the same Whole Body Phantom (Hologic) which circulated between all sites. Whole Body Phantom measurements were performed at all sites yearly, for 4 consecutive years. The percent body fat and lean body mass coefficients of variation within a given center were 0.8–1.5% and 0.7–1.5% (depending on the site), respectively. The percent body fat and lean body mass coefficients of variation between centers were 3.7% and 6.0%, respectively. The percent body fat and lean body mass coefficients of variation between the yearly scans were 0.6% and 0.9%, respectively.

Within 3–10 days after the OGTT, subjects at 4 centers were asked to return for a FSIVGTT [32], which was performed at 0800 h following an overnight fast (after 2000 h). A catheter was inserted into an antecubital vein and 3 baseline arterialized blood samples (heated box to 70°C) were obtained. At time zero glucose (300 mg/kg) was given as a smooth intravenous bolus over one minute. Insulin (0.03 units/kg) was given as an intravenous bolus 20 minutes after the start of the glucose injection. Over the 240 minutes following glucose ingestion, 22 blood samples were drawn at 2,3,4,5,6,8,10,14,19,22,24,27,30,40,50,70,90, 120,150,180,210, and 240 minutes for determination of plasma glucose and insulin concentrations.

At 8 AM on a separate day subjects from seven centers had a measurement of carotid intimal media thickness (IMT) using B-mode ultrasound (Logiq, GE Medical Systems, Milwaukee, WI) [33]. A standardized protocol was followed by all centers to assure quality control. Prior to initiation of the study, all ultrasound technologists received training at the coordinating center, directed by Dr. Howard Hodis at the University of Southern California, to ensure uniformity of measurement amongst centers. Acquisition of an image of the far wall of the common carotid was obtained and carotid IMT scans were sent to the coordinating center, where they were read blindly by two readers, as previously described [33]. In a subset of subjects at 7 of the 8 centers a second measurement was performed within 2 weeks of the initial scan to determine reproducibility and technologist performance. The coefficient of variation of these two measurements was 0.72% (range = 0.14–0.90). Carotid IMT measurements also were performed at mid study and at study end (or at time of diagnosis of diabetes) to examine the rate of change in carotid IMT.

### Recruitment and Following Visits

The first IGT subject was recruited in January of 2004 and enrollment was completed (n = 602) on February 13, 2006. All subjects were followed until they dropped out, reached the primary endpoint of diabetes, or reached the end of the blinded phase of the study 2 years from the time of recruitment of the last subject, at which time (February of 2008) all baseline measurements/procedures were repeated.

Following completion of the baseline studies, IGT subjects were randomized to receive pioglitazone, 30 mg/day, or placebo and returned 1 month later. If no adverse events were present, the dose of blinded pioglitazone (and placebo) was increased to 45 mg/day (maximum dose) and participants returned for follow-up visits at 2,4,6,8,10, and 12 months during the first year and every 3 months subsequently. On each visit a brief physical exam was performed, including measurement of weight, blood pressure, and pulse and the presence or absence of edema was recorded and graded at each visit, as was information for subjective assessment of medication side effects (excessive weight gain, edema, other). An interim medical history with a detailed cardiovascular questionnaire was obtained. Blood for determination of fasting plasma glucose concentration AST and ALT, a urine pregnancy test, and measurement of waist circumference were obtained on each follow up visit. Serum chemistries, CBC, and urine dipstick for hematuria was performed on each 6 month visit after randomization. HbA_1c _was measured every 12 months and the OGTT was repeated every 12 months. Blood for measurement of C-reactive protein, adipocytkines, markers of inflammation and other atherosclerotic cardiovascular risk factors was obtained on the final visit while on active treatment, i.e. 2 years after the last subject was enrolled, or at the time of diagnosis of diabetes. An EKG, DEXA, urine for microalbumin/creatinine ratio and 15-F_2t_-IsoP also were obtained on this final visit. Carotid IMT was measured 15–18 months after randomization and again at the final visit while on active treatment. The FSIVGTT was repeated on this final visit at centers participating in the FSIVGTT substudy.

### Conversion of IGT to Diabetes

Conversion of IGT to diabetes was made by ADA criteria [29]: (i) FPG ≥ 126 mg/dl or (ii) 2-h PG during OGTT ≥ 200 mg/dl. In either case, the diagnosis of diabetes was confirmed by an OGTT, whether the initial diagnosis was made by a FPG ≥ 126 mg/dl (i.e., on a 2–3 month follow up visit or on an annual follow up visit) or with a 2-h PG ≥ 200 mg/dl during the annual OGTT. If the repeat measurement confirmed the diagnosis of diabetes, the primary endpoint was met and a FSIVGTT/carotid IMT measurement/final visit blood draws were obtained. Any IGT subject who developed type 2 diabetes (pioglitazone-treated or placebo groups) was started on open label pioglitazone (30 mg/day and titrated to 45 mg/day [maximum dose] after one month), but the randomization code was not broken. These converters continued with all scheduled follow up visits/procedures until the study was completed (February of 2008). If the HbA_1c _or FPG increased to ≥8.0% or ≥180 mg/dl, respectively, while an open label pioglitazone, the OGTT/FSIVGTT/close out visit were performed and appropriate additional antidiabetic therapy started.

A Data Safety Monitoring Board received a copy of all adverse events, as well as detailed information about body weight, edema, AST/ALT, and other medication side effects monthly. The DSMB had a teleconference every 6 months to review all safety data. All cardiovascular events and EKG documented myocardial infarctions will be reviewed by an adjudication committee.

### Measurements

All analytical measurements were performed in a central laboratory at the Texas Diabetes Institute (San Antonio, TX). Plasma glucose concentration was measured by the glucose oxidase reaction (Glucose Oxidase Analyzer, Beckman Instruments, Fullerton, CA). Plasma insulin was measured by radioimmunoassay (Diagnostic Products, Los Angeles, CA). The interassay and intra-assay CVs for the insulin assay are 7.1% and 5.1%, respectively. Plasma C-peptide was measured by radioimmunoassay (Diagnostic Systems, Webster, TX). The interassay and intra-assay CVs for the C-peptide assay are 2.4% and 4.3%, respectively. HbA_1c _was measured by an ion-exchange HPLC instrument (Bayer DCA 2000, Leverkusen, Germany). Total plasma cholesterol and triglycerides were measured using the CHOD-DAOS method (WAKO, Richmond, VA) and an enzymatic assay (Stanbio Lab, Boerne, TX). HDL cholesterol was measured after precipitation of apolipoprotein B-containing lipoproteins with dextran sulfate-Mg^++^, using the CHOD-DAOS method (WAKO, Richmond, VA). LDL cholesterol was calculated by the Friedewald equation.

### Calculations

Incremental area under the curve (AUC) for plasma glucose and insulin concentrations during the OGTT was calculated according to the trapezoidal rule. The insulinogenic index was calculated as ΔI(AUC)/ΔG(AUC) from 0–30 and 0–120 minutes [21,22]. The insulin secretory rate during the OGTT was calculated by deconvolution of the plasma C-peptide curve [34] and expressed as ISR/ΔG [21,22,35].

During the FSIVGTT, first phase insulin secretion was calculated as the increment in plasma insulin concentration (AUC) above baseline from 0–10 minutes and as the peak increment in plasma insulin concentration (minus baseline) during the 0–10 minute time period. Indices of insulin sensitivity (S_I_) and glucose effectiveness (S_G_) were determined by minimal model analysis of insulin and glucose concentrations during the FSIVGTT as previously described [32].

The insulin secretion/insulin resistance or disposition index during the OGTT was calculated as ΔI/ΔG × Matsuda index and as ΔI/ΔG × S_I _[32,36]. The hepatic insulin resistance index was calculated as the FPI × FPG (equivalent to inverse of HOMA) [37]. The basal adipocyte insulin resistance index was calculated as the fasting plasma insulin (FPI) × fasting plasma FFA concentrations [38].

### Statistical Analysis and Sample Size

General Statistical Plan: Analysis of primary and secondary endpoints will utilize the intent-to-treat approach [39]. All participants will be included in their randomly assigned pioglitazone or placebo treatment groups. All statistical tests will be two-sided and require alpha = 0.05 to be considered significant.

#### Primary and Secondary Endpoints

The ***primary endpoint ***is the development of diabetes mellitus according to ADA criteria [29]. Accordingly, the principal analysis will be a life-table analysis of the time from randomization to the confirmed development of diabetes. Separate life-table estimated cumulative incidence curves will be calculated for the pioglitazone-treated and placebo-treated groups, which will be compared using a logrank test [38,40]. Subjects who are lost to follow up or who prematurely decide to drop out of the study will be categorized according to data obtained up to their last follow-up visit.

Secondary outcomes (i.e., cardiovascular events and mortality) will be compared using the same life-table analysis described above for the primary endpoints.

Repeated measures data (i.e., FPG, 2-hour plasma glucose during OGTT, HbA_1c_, measures of insulin secretion and insulin sensitivity, plasma lipids and other cardiovascular risk factors, blood pressure, carotid initmal media thickness, body weight and BMI, measures of fat mass and fat topography) will be compared between the pioglitazone-treated and placebo-treated groups using longitudinal data methods: (i) point prevalence of a discrete characteristic (i.e., normal or impaired glucose tolerance) at repeated visits over time [40] and (ii) multivariate rank analysis of quantitative (i.e., FPG, 2-hour plasma glucose, HbA_1c_, plasma lipids, plasma cardiovascular risk factors, etc) variables over repeated visits [40]. Differences in slopes over time (i.e., rate of change in FPG, 2-hour plasma glucose, HbA_1c_, plasma lipids, etc) between pioglitazone-treated and placebo-treated groups will be compared by the parametric linear random effects model [41].

#### Sample Size

The conversion rate of IGT to diabetes varies considerably based upon ethnicity and a number of other risk factors. The DPP [42] cited 21 studies with conversion rates (percent per year) ranging from 2.3% to 11%. In addition, they evaluated data sets from six population based cohorts [43]. The conversion rate from IGT to diabetes in these six studies was 5.8 per 100 person-years for follow-up and rose to 8.2 per 100 person-years for individuals with a fasting plasma glucose concentration > 100 mg/dl. In certain ethnic groups, (i.e., Latinos with a history of GDM), much higher conversion rates of IGT to diabetes (14.3% per year) have been reported [18,44]. The DPP [15] demonstrated a conversion rate of IGT to diabetes of approximately 11% per year. In the group of IGT subjects treated with diet/exercise and metformin, the conversion rates were decreased by 58% and 31%, respectively. In the TRIPOD study [18], GDM women treated with placebo converted to diabetes at a rate of 12.1% per year and this rate was decreased to 5.4% in the troglitazone-treated group. Based upon this information (see assumptions below), it can be calculated that approximately 600 subjects with IGT will be required to achieve 90% statistical power that pioglitazone decreases the conversion rate of IGT to type 2 diabetes by 50%. This power calculation assumes that randomized individuals drop out prior to the confirmed diagnosis of diabetes with an exponential hazard rate of 0.10 (or less) per year.

The following assumptions were used to calculate the sample size:

(i) The primary endpoint is the development of diabetes,

(ii) Participants are randomized over a 21 month period and followed for a total of 3.75 years, starting from the time that the first IGT subject is recruited,

(iii) Type I error rate (alpha) is 0.05,

(iv) The desired power is 90%,

(v) The development of diabetes in the placebo-treated group is 11% per year,

(vi) The hazard rate for the development of diabetes in the pioglitazone-treated group is reduced by 50%.

(vii) The drop out rate is 10% per year

During the course of recruitment, it became clear that the recruitment goal would not be reached in the planned 21 month period allotted for recruitment. To preserve study power, the recruitment period was lengthened 3 months to a total of 24 months to allow recruitment of the targeted number of participants with a commensurate increase in the total study duration for a follow up time of 4 years, starting from the time that the first IGT subject was recruited.

## Competing interests

RAD is on the Advisory Board of Takeda, Amylin, Eli Lilly, Roche, Novartis, Johnson and Johnson, and Bristol Meyers Squibb. RAD has Grant Support from Takeda, Amylin, Eli Lilly, Roche, Novartis, BMS, Merck, and Pfizer, he is a member of the Speakers Bureau of Takeda, Eli Lilly, and Amylin, and he is a consultant for Takeda, Amylin, Eli Lilly, Roche, Novartis, ISIS and BMS. GAB is on the Advisory Board for Amylin Pharmaceuticals and has grant support from Merck. SM has Grant Support from GSK, Sanofi-Aventis, and Intercept Pharm. RRH has grant support from Amylin, Biodel, BMS, GSK, Keryx, Lifescan, Eli Lilly, Merck, Novartis, Novo, Pfizer, Roche, Sankyo, and Veralight, he is a consultant for Amylin, Astra Zeneca, BMS, Diobex, GSK, ISIS, Eli Lilly, Merck, Novartis, Novo, Roche, Sankyo, Sanofi Aventis, and Takeda, and he is a member of the Speakers Bureau of Amylin, GSK, and Eli Lilly. NM has no conflicts of interest to declare. MAB has Research Grants from Novartis, Takeda and Pfizer, she is a consultant for BMS and Boehringer Ingleheim, and she is a speaker for Novartis, Takeda, Pfizer, Merck, and Sanofi-Aventis. RER has grant support from AstraZenica, Bayhill Therapeutics, Boehringer Ingelheim, GSK, Merck, Pfizer, Takeda, and Veralight, he is on the Advisory Board of Amylin, AstraZenica, Eli Lilly, GSK, Lifescan, NovoNordisk, Sanofi-Aventis, Takeda, and Tethys Bioscience, and he has stock ownership in Merck, Johnson & Johnson, and Abbott. FBS has no conflict of interest. AEK is on the Advisory Board for Merck, he is a member of the Speakers Bureau for Takeda, and he has grant support from Takeda and Sanofi-Aventis. DCS has grant support from Takeda. DT has grant support from Takeda. SC has no conflict of interest. TAB has grant support from Takeda and he is a member of the Speakers Bureau and on Advisory Board for Takeda. PDR has grant support from Takeda and Amylin/Lilly, and he is a member of the Speakers Bureau for Takeda and Merck.

## Authors' contributions

The initial draft of the manuscript was prepared by RAD and all authors' reviewed the manuscript and provided their comments in writing. The revised manuscript was then again reviewed by all authors for final approval.

This study was initially designed by RAD and subsequently critiqued by all authors. The study then was submitted to Takeda Pharmaceuticals NA and funded as an investigator-initiated grant. TPNA was not involved in the study design, study performance, data analyses, or manuscript preparation. At each study site, the investigators were responsible for patient recruitment and performance of all study-related procedures. All authors read and approved the final version of the manuscript.

## Pre-publication history

The pre-publication history for this paper can be accessed here:


